# L’echographie trans-oesophagienne (ETO ) à l’Institut de cardiologie d’Abidjan: indications, resultats et rentabilité diagnostique

**DOI:** 10.5830/CVJA-2015-054

**Published:** 2016

**Authors:** Jean-Baptiste Anzouan-Kacou, Christophe Konin, Charles-Philippe Zobo, Djenamba Bamba-Kamagaté, Marie-Paule N’cho-Mottoh, Bénédicte Boka

**Affiliations:** Cardiology Institute of Abidjan, Abidjan, Ivory Coast; Cardiology Institute of Abidjan, Abidjan, Ivory Coast; Cardiology Institute of Abidjan, Abidjan, Ivory Coast; Cardiology Institute of Abidjan, Abidjan, Ivory Coast; Cardiology Institute of Abidjan, Abidjan, Ivory Coast; Cardiology Institute of Abidjan, Abidjan, Ivory Coast

**Keywords:** echographie trans-oesophagienne, communication inter auriculaire, trouble du rythme, valvulopathie, rentabilité diagnostique, transoesophageal echocardiography, congenital heart disease, valvular disease, Africa

## Abstract

**Objectifs:**

Préciser les indications, les principaux résultats et la rentabilité diagnostique de l’échographie trans-oesophagienne (ETO) à l’Institut de Cardiologie d’Abidjan (ICA).

**Méthode:**

Analyse rétrospective de 103 compte-rendus d’ETO réalisés consécutivement de février 2007 à janvier 2011 à l’ICA. L’analyse a porté sur les caractéristiques d’âge, de sexe, la qualité des médecins prescripteurs, les indications et la rentabilité diagnostique (proportion de diagnostics confirmés soit le ratio anomalie retrouvée/nombre d’examen réalisés dans l’indication).

**Résultats:**

La série se composait de 47 femmes (45.6%) et de 56 hommes (54.4%) d’âge moyen 37.9 ± 16.4 ans. Les médecins prescripteurs étaient majoritairement des cardiologues (n = 57 soit 55.4%). Les indications étaient dominées par la recherche ou l’évaluation d’une communication inter-auriculaire (34.9%), la recherche de thrombus dans un trouble du rythme supra-ventriculaire avant cardioversion (18.4%), le bilan étiologique d’un accident vasculaire cérébral ischémique (13.5%) et l’évaluation d’une insuffisance mitrale (bilan lésionnel, mécanisme et/ou quantification 9.7%). Dans la recherche d’une CIA, l’ETO était contributive dans 17.3% et dans la recherche de thrombus dans 21% des cas. Aucune étiologie embolique n’a été retrouvée dans les accidents vasculaires cérébraux ischémiques. Trois examens ont été réalisés en per opératoire pour évaluation du fonctionnement des valves mécaniques et de la qualité de plasties mitrales. Aucun incident ni accident n’a été signalé au cours des 103 examens.

**Conclusion:**

Du fait du nombre élevé des cardiopathies congénitales découvertes à l’âge adulte, des troubles du rythme et des valvulopathies, l’ETO est appelée à se développer. Les indications doivent être bien posées de façon obtenir une rentabilité diagnostique optimale. Les prescriptions devraient toucher un plus grand nombre de spécialistes non cardiologues.

## Abstract (en)

**Objective:**

The aim of the study was to define the indications for and results and diagnostic accuracy of transoesophageal echocardiography (TEE) in the Abidjan Cardiology Institute. Methods: A retrospective analysis was carried out of 103 TEE reports from February 2007 to January 2011. The analysis focused on the clinical characteristics of the patients, quality of the prescribers, and indications and diagnostic accuracy (proportion of confirmed diagnoses, which is the ratio of ‘anomaly found/number of examinations made for the indication’).

**Results:**

There were 47 women (45.6%) and 56 men (54. 4%) in the study, with an average age of 37.9 ± 16.4 years. Prescribers were mostly cardiologists (n = 57; 55.4%). The indications were predominantly evaluation for atrial septal defect (ASD, 34.9%), investigation for thrombus due to rhythm disturbance before cardioversion (18.4%), aetiological evaluation of ischaemic stroke (13.5%), and assessment for mitral regurgitation (lesion assessment, mechanism and/or quantification, 9.7%). In the evaluation for an ASD, TEE was contributory in 17.3% and for thrombus, it was 21%. No embolic aetiology was found in the ischaemic strokes. Three examinations were done during cardiac surgery to assess the mechanical valves or quality of mitral plasty. There were no incidents or accidents reported during those 103 examinations.

**Conclusion:**

Because of the high number of congenital heart disease cases discovered in adulthood involving arrhythmias and valvular heart disease, TEE is likely to become more important as a means of diagnosis, and should be used correctly so as to achieve optimal diagnostic advantage. TEE should be provided by specialists not cardiologists.

## Objectifs

L’échographie trans-oesophagienne (ETO) est un examen d’imagerie diagnostique et thérapeutique indispensable en cardiologie.^1^ L’ETO est de pratique extrêmement courante en Europe et en Amérique.^1^,^2^ L’usage de cette technique semiinvasive est formalisé par de nombreuses recommandations de sociétés savantes en ce qui concerne ses indications, leur pertinence et les résultats attendus.^1^,^2^,^3^,^4^ A Abidjan (Côte d’Ivoire), cette technique est pratiquée en routine depuis 2007. L’objectif de ce travail était de préciser les indications, relever les principaux résultats et dégager la rentabilité diagnostique de l’ETO à l’Institut de Cardiologie d’Abidjan (ICA).

## Methode

Il s’agit d’une étude observationnelle et rétrospective réalisée à l’Institut de Cardiologie d’Abidjan sur une période de 48 mois, entre février 2007 et janvier 2011. Compte-rendu des patients ayant bénéficié d’une ETO à l’Institut de Cardiologie d’Abidjan. Tous les examens ont été réalisés grâce à un Echographe General Electrics (GE) Vivid 3 avec une sonde transoesophagienne multi plan 5T.

Critères d’inclusion: Comptes rendus des patients admis consécutivement pour la réalisation d’une ETO pendant la période d’étude. Critères de non inclusion: Compte-rendu incomplets du point de vue de l’âge, du sexe, des indications et/ ou de la conclusion de l’examen.

Pendant la période d’étude, 116 examens ont été réalisés. Treize examens ont été exclus, ce qui nous a permis de retenir 103 compte-rendus.

Pour chaque compte rendu, les caractéristiques d’âge, de sexe, la qualité du médecin prescripteur, l’indication de l’examen et les résultats obtenus ont été répertoriés et analysés. Les indications ont été classées par groupe nosologique. Dans chaque groupe ont été précisées les différentes pathologies et leurs proportions respectives.

Nous avons évalué la rentabilité diagnostique de l’examen comme étant la proportion de diagnostics confirmés (ratio anomalie retrouvée /nombre d’examens réalisés). Cette évaluation a été réalisée lorsque la réponse technique à la question était binaire (oui/non, présence ou absence).

## Traitement des donnees

La saisie et l’analyse des données ont été effectuées grâce aux logiciels EPI info version 6.04 et Microsoft Excel 2010. Les variables continues ont été présentées sous forme de moyenne ± écart type. Le test t de Student apparié a permis la comparaison des moyennes avec un seuil de significativité statistique à 0.05.

## Resultats

L’âge moyen de l’ensemble des 103 patients était de 37.9 ± 16.4 ans. La série se composait 47 femmes (45.6%) et de 56 hommes (54.4%) avec un sex ratio H/F de 1.19. La répartition de la qualité des médecins prescripteurs était la suivante: cardiologues (n = 57 soit 55.4%), cardio-pédiatres (n = 29, soit 19.4%), neurologues (n = 10 soit 9.7%), chirurgiens cardiaques (n = 10 soit 9.7%), internistes (n = 6 soit 5.8%).

Les examens ont été divisés en 2 grands groupes: examens demandés en dehors d’un contexte chirurgical aigu (n = 99 soit 96.1%); examens demandés en contexte péri-opératoire de chirurgie cardiaque programmée (n = 4 soit 3.9%).

## Examens demandés en dehors d’un contexte chirurgical aigu (n = 99)

Les indications retrouvées étaient:

la recherche ou l’évaluation d’une cardiopathie congénitale (n = 40 soit 40.4%)
l’évaluation d’une valvulopathie (n = 24 soit 24.2%)
le bilan de troubles du rythme supra ventriculaires (TDR) (n = 19 soit 19.2%)
le bilan étiologique d’un accident vasculaire cérébral ischémique (AVCI) (n = 14 soit 14.2%)
la recherche de masse intra-auriculaire gauche suspectée à l’échographie trans – thoracique (n = 1 soit 1%)
la suspicion de dissection aortique (n =1 soit 1%)

Les détails des indications dans le groupe des cardiopathies congénitales apparaissent dans le Table 1. L’évaluation dans le cadre de communications inter-auriculaires (CIA) représentait 36 examens soit 90% des examens demandés pour cardiopathie congénitale et 34.9% (36/103) de l’ensemble des indications. L’aide au diagnostic positif d’une CIA constituait la principale question technique.

**Tableau 1:** Détail des indications dans la recherche et l’évaluation des cardiopathies congénitales (n = 40)

*Indications*	*n*	*%*
Recherche de CIA, ETT non-contributive	29	72.5
CIA connue, bilan anatomique	3	7.5
Réévaluation d’une CIA	2	5
Bilan pré cathétérisme d’une CIA	1	2.5
CIA opérée, dilatation des cavités droites	1	2.5
CIV connue, anatomie d’une membrane sous aortique	2	5
Recherche de CIV	1	2.5
CIV opérée, appréciation d’un shunt résiduel sur patch	1	2.5
Total	40	100

Les communications inter-ventriculaires (CIV) représentaient 4 examens soit 10% des examens demandés pour cardiopathie congénitale et 3.9% (4/103) de l’ensemble des indications.

Les détails des indications dans le groupe des valvulopathies apparaissent dans le Table 2.. L’évaluation d’une insuffisance mitrale (quantification et/ou bilan lésionnel) représentait 10 examens soit 41.7% des examens demandés pour valvulopathie et 9.7% (10/103) de l’ensemble des indications.

**Tableau 2:** Détail des indications dans l’évaluation des valvulopathies acquises (n = 24)

*Indication*	*n*	*%*
Insuffisance mitrale sévère: anatomie valvulaire et sous valvulaire	8	33.3
Quantification d’une insuffisance mitrale	1	4.2
Analyse d’une insuffisance mitrale par perforation mitrale	1	4.2
Rétrécissement mitral: anatomie valvulaire et sous valvulaire	3	12.5
Insuffisance aortique: recherche de mécanisme	4	16.6
Suspicion d’endocardite infectieuse: recherche de végétations	5	20.9
Suspicion de dysfonction de prothèse mécanique	2	8.3
Total	24	100

Parmi les 5 demandes pour suspicion d’endocardite infectieuse Table 2., il s’agissait 4 fois de recherche de végétation mitrale, 1 fois de recherche de végétation tricuspidienne, et 1 fois de recherche de végétation sur prothèse mécanique en position mitrale. Dans le groupe des troubles du rythme (19/103 soit 18.4% du total des indications), la recherche de thrombus avant cardioversion pour arythmie complète par fibrillation auriculaire représentait 16 demandes soit 84.2% des examens demandés pour TDR et 15.5% (16/103) de l’ensemble des indications.

La recherche de thrombus avant cardioversion pour flutter auriculaire représentait 3 demandes soit 15.8% des indications pour TDR. Le bilan étiologique d’un accident vasculaire cérébral AVCI représentait 14/103 soit 13.5% de l’ensemble des indications. Le Table 3. permet de récapituler la rentabilité diagnostique de l’ETO en fonction des indications, lorsque la réponse technique à la question était binaire (oui/non, présence ou absence). Cette analyse a concerné 73 examens.

**Tableau 3:** Rentabilité diagnostique en fonction de l’indication

*Indications (n = 73)*	*Proportion sur l’ensemble des demandes d’examen (%)*	*Rentabilité diagnostique (%)*
Recherche de CIA, ETT non-contributive (n = 29)	28.1	17.3
Recherche d’une CIV (n = 1)	0.9	50
CIV opérée, appréciation du shunt résiduel sur patch (n = 1)	0.9	100
Suspicion d’endocardite infectieuse: recherche de végétations (n = 5)	4.8	20
Recherche de dysfonction de prothèse mécanique (n = 2)	1.8	50
AC/FA, et flutter auriculaire recherche de thrombus (n = 19)	3.9	21
Recherche de MIAG (n = 1)	0.9	0
Recherche de dissection aortique (n = 1)	0.9	100
Recherche d’une cause cardiaque d’embolie dans le cadre d’un AVC ischémique (n = 14)	13.6	0

## Examens demandés en contexte chirurgical aigu

Trois patients ont bénéficié d’une ETO per opératoire. Il s’agissait dans tous les cas de valvulopathies opérées soit par remplacement valvulaire soit par plastie. Les ETO étaient indiquées dans tous les cas pour évaluation du fonctionnement des valves mécaniques et de la qualité de plasties.

Une patiente a bénéficié d’une ETO en post-opératoire immédiat pour établissement du mécanisme d’un état de choc. Aucun incident ni accident n’a été signalé au cours des 103 examens.

## Discussion

Notre travail permet de rapporter l’activité d’ETO à l’ICA, d’en préciser les principales indications et la rentabilité diagnostique en fonction des indications. Les indications étaient dominées par la recherche ou l’évaluation d’une communication interauriculaire (34.9% de l’ensemble des indications), la recherche de thrombus dans un trouble du rythme supra-ventriculaire avant cardioversion (18.4% de l’ensemble des indications), le bilan étiologique d’un AVC ischémique (13.5 % de l’ensemble des indications) et l’évaluation d’une insuffisance mitrale (bilan lésionnel, mécanisme et/ou quantification 9.7% de l’ensemble des indications). La plupart des examens (96.1%) étaient réalisés en dehors du contexte aigu de chirurgie cardiaque programmée.

Dans la littérature médicale en Afrique sub-saharienne, en dehors de l’Afrique du Sud, il est fait très peu mention des activités d’ETO. Une étude gabonaise 5 rapporte une expérience de 146 examens sur 81 mois, de 2000 à 2006. Les examens ont été réalisés grâce à une sonde monoplan, donc de technologie plus ancienne. Les patients ont un âge moyen de 55 ans (plus élevé que celui des patients de notre série qui était de 27.9 ans).

Une équipe béninoise rapporte 24 ETO sur une période de 29 mois 5 dans l’indication unique de l’aide à la stratégie de réduction d’une fibrillation auriculaire. Cette indication représentait 16 demandes soit 15.5% des indications dans notre série. Des auteurs Dakarois 6 rapportent 33 cas d’examens per-opératoires dans le cas de plasties mitrales, indication retrouvée 4 fois dans notre pratique.

Dans l’expérience gabonaise,^7^ les indications se répartissent différemment: recherche étiologique dans les AVC ischémiques (37.6%), recherche de mécanisme de valvulopathie avant chirurgie réparatrice (20.5%), aide à la stratégie de réduction de troubles du rythme (16.4%), recherche d’endocardite infectieuse 11.6%. Dans cette série aucun examen n’a été réalisé dans l’indication d’une cardiopathie congénitale.

Dans une série américaine récente, évaluant la pertinence des indications de 202 ETO réalisées sur une période de 3 mois,^4^ les 3 indications principales étaient l’aide à la stratégie de réduction des troubles du rythme (49%), la recherche d’endocardite infectieuse (13%) et les procédures de cardiologie interventionnelle non coronariennes (9%). Il faut donc conclure que la pratique des examens est liée non seulement au spectre des cardiopathies dans le milieu de travail, mais certainement à l’orientation des pratiques par établissement.

Les spécialistes en cardiologie (cardiologues 55.4%, cardiopédiatres 19.4%, chirurgiens cardiaques 9.7%), représentaient les principaux prescripteurs des examens, comme dans la plupart des séries.^3^,^4^ Les neurologues représentent pour Mansour aux Etats-unis^3^ 8.9% des prescripteurs, à l’identique de nos résultats.

L’ETO a des avantages certains sur l’ETT du fait de l’utilisation de sondes de haute fréquence à proximité des structures cardiaques, avec une résolution élevée des images.^8^,^9^ Evaluer la rentabilité diagnostique est importante du fait du caractère semiinvasif de l’ETO et donc non dénué de complications, posant le problème de la pertinence des indications et des résultats attendus.^3^,^4^ Dans notre série nous n’avons noté aucun incident. Les complications sérieuses de l’ETO sont rarissimes, estimées à 1/10 000 examens.^10^

La rentabilité des examens est variable en fonction de l’indication. L’examen a été le plus rentable dans la recherche de dissection aortique et dans la recherche d’un shunt résiduel sur patch de CIV (100%, Table 3.). Cependant ces indications ne comptaient chacune qu’un seul examen. Dans la recherche de CIA (29 demandes soit 28.1% des demandes sur l’ensemble des indications, Table 3.), l’ETO n’a mis en évidence l’anomalie recherchée que dans 17.3% des cas. La justesse de l’indication conditionne la qualité du résultat. A l’ETT dans la coupe apicale 4 cavités il apparait un semblant de défaut dans un septum normal, donnant l’illusion d’une fausse CIA, lié au faible signal écho reflété par le septum inter-auriculaire.^11^ La faible rentabilité diagnostique de l’ETO dans les CIA dans notre série pourrait s’expliquer par des indications mal posées.

Dans l’endocardite infectieuse (4.8% des indications) la rentabilité diagnostique était de 20%. La supériorité de l’ETO sur l’ETT dans le domaine de l’endocardite infectieuse est bien établie, les critères échographiques faisant partie des critères diagnostiques.^12^ L’ETO est particulièrement intéressante pour la mise en évidence des végétations de petite dimension, inférieures à 5 mm, et offre ainsi la possibilité d’un diagnostic plus précoce de l’endocardite, permettant une mise en route rapide du traitement.^12^ La sensibilité de l’ETO sur une série de 96 patients dans la détection des végétations serait de 100% contre 63% pour l’ETT.^13^ Malgré cette faible rentabilité, toute suspicion d’endocardite infectieuse doit faire réaliser une ETO devant une ETT non-contributive.^12^ Dans l’indication d’un AVCI dans notre série aucun examen n’a permis de retrouver des arguments pour une cardiopathie emboligène. La taille de notre échantillon pourrait aisément l’expliquer. Ailleurs,^14^,^15^ dans les AVCI, chez 15 à 25% des patients, l’ETO permet de mettre en évidence une source cardiaque d’embolie.

## Conclusion

L’ETO est un examen indispensable dans l’aide au diagnostic et à l’évaluation de plusieurs affections cardiovasculaires. Les indications doivent être bien posées de façon obtenir une rentabilité diagnostique optimale notamment dans les communications inter-auriculaires. Les prescriptions devraient provenir d’un plus grand nombre de spécialistes non cardiologues, notamment les neurologues, du fait de l’importance des AVCI dans notre milieu.
